# Formulation of the Menu of a General Hospital After Its Conversion to a “COVID Hospital”: A Nutrient Analysis of 28-Day Menus

**DOI:** 10.3389/fnut.2022.833628

**Published:** 2022-04-13

**Authors:** Paraskevi Detopoulou, Zena Hardan Al-Khelefawi, Garifallia Kalonarchi, Vasilios Papamikos

**Affiliations:** ^1^Department of Nutrition, “Korgialenio-Benakio” Hellenic Red Cross Hospital, Athens, Greece; ^2^Department of Nutrition and Dietetics, University of Peloponnese, Kalamata, Greece; ^3^Department of Dietetics and Nutrition, Harokopio University, Athens, Greece

**Keywords:** hospital menu, COVID-19, malnutrition, energy, protein

## Abstract

**Aim:**

The aim of the present study was to modify the hospital menu to increase energy and protein provision in COVID-19 patients.

**Methods:**

After the conversion of our hospital to a COVID-19 hospital, eggs, and comfort foods such as vanilla pudding were added to the menu to boost energy and protein intake of patients. All meals of the standard menu of the hospital, i.e., breakfast, lunch, and dinner were recorded for 14 consecutive days during two periods: pre-COVID-19 and after being converted to a “COVID hospital.” The menus were analyzed with the use of the USDA database.

**Results:**

The total content of energy (1,873 ± 87 vs. 2,489 ± 137 Kcal), protein (97 ± 11 vs. 126.4 ± 18.7 g), fat (55 ± 9 vs. 74.1 ± 12.8 g), and carbohydrate (241.0 ± 16.0 vs. 323.0 ± 16.0 g) of the provided menus was increased in the COVID-19 period compared to the pre-COVID period. The leucine provision was also increased (4.8 ± 1.08 vs. 7.2 ± 1.3 g). Changes in protein and leucine were greater for breakfast (10 vs. 21 g protein and 0.8 vs. 1.7 g of leucine). The menu during COVID-29 provided more vitamin C (69.5 vs. 109.4 mg), thiamine (1.5 vs. 1.6 mg), riboflavin (2.1 vs. 2.6 mg), niacin (20.6 vs. 27.2 mg), pantothenic Acid (5.7 vs. 7.9 mg), vitamin B6 (2 vs. 2.6 mg), folate (274 vs. 334 μg), B**_12_** (4.8 vs. 6.2 μg), choline (296 vs. 458 mg) as well as vitamins A (8,564 vs. 21,258 IU), D (3.9 vs. 4.7 μg), and K (59.3-111.5 μg). As far as micronutrients are concerned, the provisions of calcium (972 vs. 1375 mg), iron (10.2-12.8 mg), magnesium (236 vs. 294 mg), phosphorus (1,325 vs. 1,807 mg), copper (1.0 vs. 1.3 mg), manganese (2.1 vs. 2.4 mg) and selenium (148 vs. 183 μg) were increased during the COVID-19 period.

**Conclusion:**

Simple menu changes and addition of comfort foods can substantially boost the nutrient content of a hospital diet, which in concert with provision of oral nutritional supplements could have an impact on patients’ nutritional status.

## Introduction

The role of nutrition in disease onset and therapy is critical (1) and its role in the COVID-19 epidemic has been stressed by researchers (2) and international bodies (3–5). Hospitalized patients with COVID-19, in particular, are characterized by an inflammatory state (6) and are at increased nutritional risk, which in turn is connected to disease severity, mortality (7, 8) and prolonged hospital stay (9).

The impairment of nutritional status has multiple etiologies related to the disease *per se* as well the reduced nutrient intake or increased losses of patients (3). More particularly, fever and respiratory distress increase hydration and energy needs of COVID-19 patients, while isolation and inactivity may lead to sarcopenia (10, 11). Moreover, shortness of breath, nausea, dry mouth as well as loss of smell and taste may render oral intake difficult (12). As a result, patients report a decreased appetite and a feeling of being full (10). In parallel, they have limited access to snacks since visitors are not allowed (13), who in other circumstances could significantly contribute to an increase in patient’s energy intake (14). In patients presenting with diarrhea, nutritional status may further worsen (15).

At the same time, energy and protein intake is important in hospitalized patients in order to maintain muscle mass, ensure the function of vital organ systems and facilitate rehabilitation (16). The enteral route is associated with better outcomes compared to the parenteral route (17). ESPEN recommends 27–30 Kcal/kg, more than 1 g/kg of protein and a ratio of energy provided by fat and carbohydrates ranging from 30:70 for subjects with no respiratory deficiency, to 50:50 for ventilated patients (3). Other official recommendations also highlight the need for energy and protein intake (18). Moreover, oral nutritional supplements are recommended to achieve the targets of energy and protein (3). The British Dietetic Association encourages a “food first” approach, which is based on the consumption of ordinary foods to improve nutritional status (19). Therefore, managing the nutritional intake of hospitalized patients with COVID-19 through modified menus and oral nutritional supplements, when needed, is a crucial point in the nutritional management of disease (3). The data regarding hospital nutritional management of COVID-19 in Greece are scarce and more focused in critically ill patients (17, 20).

In the current work, we present the changes in the standard menu of a Greek state hospital to better comply with the requirements of COVID-19 patients, after its conversion to a “COVID hospital.”

## Materials and Methods

All meals of the standard menu of the hospital i.e., breakfast, lunch, and dinner were recorded for 14 consecutive days during two periods: pre-COVID-19 and after being converted to a “COVID hospital.” The changes were made by the Clinical Nutrition Unit to increase energy and protein intake with comfort foods. The scientific board of our hospital approved the rational of this study.

It is noted that menus being part of a specialized diet (i.e., diet to combat diarrhea, liquid diet etc.) were not considered in the present analysis. Blenderized diets were also excluded, since they substantially differentiate in nutrient content from regular diets (21).

The food-based changes are further illustrated through a nutrient analysis approach. The two sets of 14-day menus (Monday to Sunday) were analyzed using the USDA database and the corresponding values of containing macro- and micro- nutrients for each day were obtained (22). More particularly the following data was obtained: energy, protein, leucine, fat, saturated fat, monounsaturated fat, polyunsaturated fat, carbohydrate, fiber, sugar, calcium, iron, magnesium, phosphorus, zinc, copper, manganese, selenium, vitamin C, thiamine, riboflavin, niacin, pantothenic acid, vitamin B6, folate, choline, vitamin B_12_, vitamin A, vitamin E, Vitamin D, and vitamin K. Food portions were based on standardized quantities of foods used in our hospital for each recipe. Weight losses with cooking were calculated after weighting raw and cooked foods and were in line with the retention factors published by the USDA (23).

Normality was tested with the Kolmogorov-Smirnoff criterion. Normally distributed continuous variables are presented as mean values ± standard deviation, while skewed variables as median and 25th–75th quartiles. *T*-test or Mann-Whitney test was applied for comparisons of parametric or non-parametric variables, respectively. SPSS program was used for statistical analysis version 19, release 19.0.01 (IBM Hellas, Chalandri, Greece).

## Results

**FIGURE 1:**
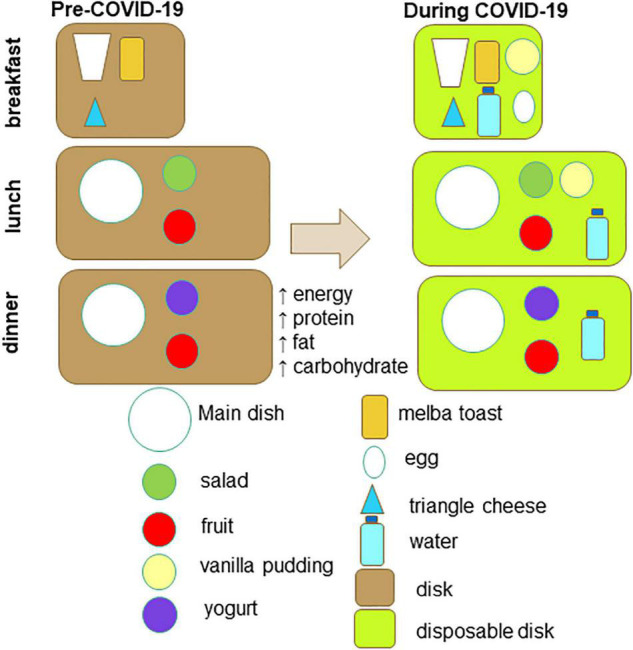
Graphical presentation of the menu changes.

The menus of the pre-COVID-19 and COVID-19 period are displayed on Table 1. Several changes were made at breakfast and main meals. As far as the breakfast is concerned, at the pre-COVID-19 period it included a triangle cheese, melba toasts and milk (or tea/chamomile). At the COVID-19 period an egg and a vanilla pudding were added. Moreover, a vanilla pudding or a rice milk pudding was added mostly at lunch (Table 1 and Figure 1). Table 2 indicates the daily or weekly food frequencies of the meals provided along with the Greek recommendations (24), so that comparisons can be made. As it is shown, both menus were in line with the fish recommendations, but provided fewer portions of fruits, vegetables, oils, and legumes than those recommended. Moreover, both menus provided more portions of grains, meat, and poultry while the COVID-19 menu provided more eggs and dairy products than those recommended for the healthy population.

**TABLE 1:** Description of the standard hospital meals and the hospital meals for COVID patients.

			Energy (Kcal)	Protein (g,% energy)	HBV protein (g,% of meal protein)	Leucine (g)	Carbohydrates (g/% meal energy)	Fat (g/% meal energy)
All days breakfast	Pre-COVID	Milk (or Tea or Chamomile) 1 packet Sugar 1 Cheese Triangle 2 pcs. Melba Toasts	256	10.0 15.7%	8.2 82.5%	0.81	31 48.5%	7.1 25.1%
	Post-COVID	Milk (or Tea or Chamomile) 1 packet Sugar 1 Cheese Triangle 1 boiled egg 2 pcs. Melba Toasts Vanilla pudding	537	21.3 15.9%	19.5 91.7%	1.7	61.8 46%	19.3 32.4%
1st day lunch	Pre-COVID	Spaghetti Bolognese Cucumber Apple White bread	715	32.44 18.1%	19.31 59.5%	2.0	97.7 54.6%	21.5 27.1%
	Post COVID	Spaghetti Bolognese Grated cheese Cabbage and carrot salad Apple Rice pudding White bread	1,022	43.9 17.2%	29.28 66.6%	2.2	142.4 55.7%	31.0 27.3%
Dinner	Pre-COVID	Chicken with lemon sauce Mashed potatoes Apple Yogurt White bread	1,004	57.1 22.7%	48.6 85.1%	2.4	96.1 38.3%	43.3 38.8%
	Post COVID	Chicken with lemon sauce Roasted potatoes Apple Yogurt White bread	1,020	57.6 22.6%	46.7 81.1%	2.4	122.7 48.1%	33.4 29.5%
2nd day lunch	Pre-COVID	Fish with lemon sauce Potato salad Boiled zucchini Apple White bread	760	39.4 20.7%	24.9 63.3%	2.5	129.6 68.1%	10.9 12.9%
	Post COVID	Grilled fish Potato salad Cabbage and carrot salad Apple Vanilla pudding White bread	930	43.3 18.6%	29.9 69.1%	2.9	164.7 70.8%	12.4 12.0%
Dinner	Pre-COVID	Lasagna Tomato sauce Grated cheese Apple Yogurt White bread	785	37.1 18.9%	22.7 61.2%	1.0	110.6 56.3%	22.3 25.5%
	Post COVID	Spaghetti Tomato sauce with vegetables (zucchini-carrot -peppers) Grated cheese Apple Yogurt White bread	796	37.5 18.8%	22.7 60.6%	1.1	113.2 56.8%	22.4 25.3%
3rd day lunch	Pre-COVID	Pork with mustard sauce Rice Pilaf Apple Cucumber White bread	749	45.3 24.2%	35.1 77.4%	3.4	102.4 54.7%	16.9 20.3%
	Post COVID	Pork with lemon sauce Roasted potatoes Cabbage and carrot salad Apple Vanilla pudding White bread	1,025	53.2 20.7%	40.1 75.4%	3.8	160.3 62.5%	19.9 17.5%
Dinner	Pre-COVID	Potato ragù Yogurt Apple White bread	736	27.5 14.9%	18 65.3%	0.026	103.5 56.2%	24.8 30.3%
	Post COVID	Potatoes ragù with mushrooms Feta cheese Apple Yogurt White bread	852	36.2 17.0%	25.1 69.3%	0.7	111.4 52.3%	31.1 32.8%
4tb day lunch	Pre-COVID	Braised beef with Tomato sauce Orzo/Risoni Apple Cucumber White bread	870	55.22 25.37%	42.09 76.22%	3.9715	97.77 44.92%	28.9 29.88%
	Post COVID	Braised beef with tomato sauce Orzo/Risoni Grated cheese Apple Rice pudding Cabbage and carrot salad White bread	1,089	66.75 24.51%	52.06 77.99%	4.546	142.56 52.35%	28.99 23.95%
Dinner	Pre-COVID	Rice Pilaf with carrots, peas and turkey pieces Apple Yogurt White bread	851	51.04 23.98%	41.25 80.82%	1.975	105.37 49.52%	24.43 25.83%
	Post COVID	Chicken with lemon sauce Rice Pilaf Apple Yogurt White bread	998	56.353 22.58%	46.76 82.98%	2.466	106.82 42.80%	37.574 33.87%
5th day lunch	Pre-COVID	Pork with peppers Mashed potatoes Apple Cucumber White bread	740	45.82 24.76%	37.04 80.84%	3.3844	88.64 47.90%	22.563 27.43%
	Post COVID	Pork with Peppers mashed potatoes Apple Cabbage and carrot salad Vanilla pudding White bread	994	52.38 21.07%	42.04 80.26%	3.885	130.59 52.54%	29.693 26.88%
Dinner	Pre-COVID	Mixed veg. ragù “Tourlou” (Potato, zucchini, Carrot, eggplant) Yogurt Apple White bread	789	28.76 14.57%	18 62.59%	0.34405	121.91 61.77%	22.15 25.25%
	Post COVID	Rice Pilaf with vegetables (Carrot, Peas, zucchini, peppers) and sauce and turkey pieces Apple Yogurt White bread	1,060	93.093 35.12%	82.57 88.70%	4.853	111.04 41.90%	25.594 21.73%
6th day lunch	Pre-COVID	Chicken braised with tomato sauce Lasagna Apple Cucumber White bread	824	41.89 20.33%	28.76 68.66%	2.671	97.77 47.44%	28.93 31.58%
	Post COVID	Chicken braised with tomato sauce Rice Pilaf with carrots and peas Apple Yogurt Cabbage and carrot salad Vanilla pudding White bread	1,261	63.76 20.22%	51.76 81.18%	2.985	150.95 47.88%	44.67 31.88%
Dinner	Pre-COVID	Rice Pilaf with mixed vegetables (Carrot, Peas) and turkey pieces Yogurt Apple White bread	807	41.89 20.33%	28.76 68.66%	2.671	97.77 47.44%	28.93 31.58%
	Post COVID	Spaghetti with white sauce and bacon Grated cheese Apple Rice pudding White bread	896	30.443 13.59%	17.54 57.62%	1.708	132.33 59.07%	27.574 27.69%
7th day lunch	Pre-COVID	Fish with lemon sauce Potato salad Apple Boiled zucchini White bread	760	39.443 20.74%	24.99 63.36%	2.5735	129.67 68.17%	10.974 12.98%
	Post COVID	Fish with lemon sauce potato, zucchini, carrot Apple Rice pudding Cabbage and carrot salad White bread	968	43.813 18.10%	30.19 68.91%	2.721	165.91 68.53%	15.594 14.49%
Dinner	Pre-COVID	Omelet (Pepper, potato, zucchini) Feta cheese Yogurt Apple White bread	994	48.31 19.44%	38.34 79.36%	2.1211	113.72 45.75%	39.31 35.59%
	Post COVID	Rice Pilaf with (Carrot, Peas, zucchini, peppers) and turkey pieces Apple Yogurt White bread	813	51.35 25.26%	41.25 80.33%	1.988	106.77 52.53%	19.82 21.94%
8th day lunch	Pre-COVID	Spaghetti Bolognese Apple Cucumber White bread	715.2	32.44 18.14%	19.31 59.53%	1.713625	97.7 54.64%	21.54 27.11%
	Post COVID	Spaghetti Bolognese Grated cheese Apple Rice pudding Cabbage and carrot salad White bread	1,022	43.97 17.21%	29.28 66.59%	2.288	142.56 55.79%	31.01 27.30%
Dinner	Pre-COVID	Chicken with lemon sauce Mashed potatoes Yogurt Apple White bread	1,004	57.113 22.75%	48.65 85.18%	2.4926	96.13 38.29%	43.337 38.83%
	Post COVID	Chicken with lemon sauce mashed potatoes Apple Yogurt White bread	1,004	57.113 22.75%	48.65 85.18%	2.493	96.13 38.29%	43.337 38.83%
9th day lunch	Pre-COVID	Fish with lemon sauce Potato salad Apple White bread	767	36.593 19.07%	24.99 68.29%	2.481	122.97 64.08%	14.764 17.31%
	Post COVID	Fish with lemon sauce Potatoes, zucchinis, carrots Apple Rice pudding Cabbage and carrot salad White bread	968	43.813 18.10%	30.19 68.91%	2.576	165.91 68.53%	15.594 14.49%
Dinner	Pre-COVID	Lasagna Tomato sauce Grated cheese Yogurt Apple White bread	785.2	37.15 18.93%	22.77 61.29%	1.0925	110.64 56.36%	22.32 25.58%
	Post COVID	Rice Pilaf with carrots, peas and turkey pieces Yogurt Apple White bread	1,060	93.093 35.12%	82.57 88.70%	4.853	111.04 41.90%	25.594 21.73%
10th day lunch	Pre-COVID	Pork with mustard sauce rice Pilaf Apple Cucumber White bread	749	45.393 24.23%	35.15 77.43%	3.4036	102.49 54.71%	16.964 20.37%
	Post COVID	Pork with peppers Mashed potatoes Apple Cucumber White bread	1,033	46.39 17.96%	33.76 72.77%	2.939	156.5 60.60%	25.81 22.49%
Dinner	Pre-COVID	Omelet (Zucchini, pepper, potato) Yogurt Apple White bread	862	41.2 19.11%	31.23 75.80%	1.421	111.78 51.86%	28.67 29.93%
	Post COVID	Spaghetti Tomato sauce with peppers and carrots Grated cheese Yogurt	796	37.57 18.87%	22.77 60.61%	1.118	113.2 56.87%	22.43 25.35%
11th day lunch	Pre-COVID	Braised beef with tomato sauce Orzo/Risoni Apple Cucumber White bread	818	55.23 27.00%	42.09 76.21%	3.9715	97.8 47.81%	23.56 25.92%
	Post COVID	Spaghetti Minced meat with sauce Grated cheese Apple Cabbage and carrot salad Vanilla pudding White bread	1,027	43.77 17.04%	29.08 66.44%	2.675	140.19 54.59%	32.71 28.66%
Dinner	Pre-COVID	Rice Pilaf with carrots, peas and turkey pieces Yogurt Apple White bread	851	51.04 23.98%	41.25 80.82%	1.975	105.37 49.52%	24.43 25.83%
	Post COVID	Chicken with lemon sauce Roasted potatoes Apple Yogurt White bread	1,020	57.603 22.58%	46.76 81.18%	2.46	122.71 48.11%	33.444 29.50%
12th day lunch	Pre-COVID	Pork with peppers Mashed potatoes Apple Cucumber White bread	740	45.82 24.76%	37.04 80.84%	3.384	88.64 47.90%	22.563 27.43%
	Post COVID	Pork with Peppers Mashed potatoes Apple Cabbage and carrot salad Vanilla pudding White bread	928	50.17 21.62%	42.04 83.80%	3.742	115.21 49.65%	29.353 28.46%
Dinner	Pre-COVID	Spaghetti Tomato sauce with peppers and carrots Grated cheese Yogurt Apple White bread	794	37.4 18.84%	22.77 60.88%	1.112	112.8 56.81%	22.38 25.36%
	Post COVID	Rice Pilaf with vegetables (Carrot, Peas, zucchini, peppers) and sauce and turkey pieces Apple Yogurt White bread	837	52.413 25.04%	41.89 79.92%	2.039	111.04 53.06%	20.214 28.46%
13th day lunch	Pre-COVID	Chicken braised with tomato sauce Orzo/Risoni Apple Cucumber White bread	780	41.89 21.48%	28.76 68.66%	2.663	97.77 50.13%	24.24 27.96%
	Post COVID	Chicken braised with tomato sauce Rice Pilaf with Carrot, Peas Apple Cabbage and carrot salad Vanilla pudding White bread	1,067	45.76 17.15%	33.76 73.78%	2.985	142.99 53.59%	34.67 29.24%
Dinner	Pre-COVID	Rice Pilaf with mixed vegetables (Carrots, peas) and turkey pieces Yogurt Apple White bread	807	51.04 25.30%	41.25 80.82%	1.975	105.37 52.23%	19.74 22.01%
	Post COVID	Pork with lemon sauce Roasted potatoes Apple Yogurt Vanilla pudding White bread	950	63.993 26.93%	53.15 83.06%	3.369	122.71 51.65%	22.724 21.52%
14th day lunch	Pre-COVID	Fish with lemon sauce Potato salad Apple Boiled zucchini White bread	760	39.443 20.74%	24.99 63.36%	2.772	129.67 68.17%	10.974 12.98%
	Post COVID	Orzo/Risoni Octopus boiled Apple Cabbage and carrot salad Vanilla pudding White bread	1,037	68.89 26.57%	54.2 78.68%	4.549	146.98 56.68%	18.64 16.17%
Dinner	Pre-COVID	Omelet (spinach, zucchini) Feta cheese Yogurt Apple White bread	807	45.3 22.43%	37.71 83.25%	1.940	71.31 35.32%	38.49 42.89%
	Post COVID	Potato ragù (Mushrooms, potatoes) Feta cheese Apple Yogurt White bread	852	36.2 17.00%	25.11 69.36%	0.792	111.44 52.32%	31.14 32.89%

*Water was also provided with every meal. HBV, High Biological Value.*

**TABLE 2:** Daily or weekly frequencies of foods provided and comparison with Greek recommendations of the healthy population.

Food groups	Pre-COVID	Post-COVID	Greek recommendations for healthy adults	Portion
Vegetables	1.2 ↓	1.2 ↓	4 portions/day	150–200 g
Fruits	2 ↓	2 ↓	3 portions/day	120–200 g
Grains, rice, potatoes	9 ↑	9 ↑	5- 8 portions/day	1 slice of bread (30 g), 2 Melba toasts, 1/2 cup pasta/rice (70–90 g)
Dairy	2.3	4.2 ↑	2 portions/day	1 cup milk, 200 g yogurt, 30 g cheese
Legumes	0 ↓	0 ↓	3 portions/day	1 cup
Meat	3.5 ↑	7 ↑	Up to 1 time/week	120–150 g
Poultry	3 ↑	4.5 ↑	1–2 portions/week	120–150 g
Eggs	4	7 ↑	Up to 4/week	1 egg
Fish	2	2	2–3 portions/week	150 g
Oils	1 ↓	1↓	4–5 portions/day	1 Tbs olive oil

The menu changes in the COVID-19 period led to differentiations concerning energy, which increased from 1,873 ± 87 Kcal to 2,489 ± 137 Kcal (*p* < 0.001). Moreover, the absolute values (in grams) of protein, carbohydrate, and fat provided were increased, whereas changes were evident in some micronutrients (Table 3). The provided leucine was increased, and the highest protein and leucine increase was noted for breakfast, in which protein and leucine provision was doubled (10 vs. 21 g protein and 0.8 vs. 1.7 g of leucine) (Table 1). The total percentages of macronutrients were not different before and during the COVID-19 period. More particularly, in the period before COVID-19 the hospital menu consisted of 21% protein, 26% fat and 51.4% carbohydrates while during the COVID-19 period it consisted of 20% protein, 27% fat and 52% carbohydrates (rounded values). The menu during COVID-19 provided more vitamin C, B-vitamins as well as vitamins A, D, and K. As far as micronutrients are concerned, the provisions of calcium, iron, magnesium, phosphorus, copper, manganese, and selenium were increased during the COVID-19 period.

**TABLE 3:** Energy, macro, and micro-nutrient content of the menu provided before and after the conversion of our hospital to a COVID hospital.

				Before COVID-19		After COVID-19		*P*-value
Energy and nutrients	RDA or AI* (F)	RDA of AI* (M)	ESPEN recommendations for COVID-19 patients	Mean or median	*SD* or 25th–75th	Mean or median	*SD* or 25th–75th	
Energy (Kcal)			27–30 Kcal/kg	1,873	87	2489.2	137.1	<0.001
Protein (g)	46	56	1 g/kg or more	97	11	126.4	18.7	<0.001
Leucine (g)	2.9*	2.9*		4.8	1.08	7.2	1.3	<0.001
Fat (g)	30–40%	30–40%		55	9	74.1	12.8	<0.001
Saturated fat (g)				22	3	42.9	6.1	<0.001
Monounsaturated fat (g)				24	4	33.3	5.9	<0.001
Polyunsaturated fat (g)				9	2	11.4	1.8	<0.05
Carbohydrate (g)	130	130		241.0	16.0	323.0	16.0	<0.001
Fiber (g)	21	30		21.0	3.0	25.0	2.0	<0.001
Sugar (g)				85.7	5.4	146.0	7.0	<0.001
Calcium (mg)	1,200	1,000		972.7	159.7	1375.4	125.7	<0.001
Iron (mg)	8	8		10.2	1.2	12.8	3.8	<0.001
Magnesium (mg)	320	420		236.8	50.4	294.6	36.4	<0.001
Phosphorus (mg)	700	700		1325.8	201.6	1807.0	144.5	<0.001
Zinc (mg)	8	11		10.0	4.1	12.8	3.3	0.06
Copper (mg)	0.9	0.9		1.0	0.3	1.3	0.4	0.05
Manganese (mg)	1.8	2.3		2.1	1.7–2.3	2.4	2.0–2.6	<0.001
Selenium (μg)	55	55		148.0	16.0	183.0	34.6	0.05
Vitamin C (mg)	175	90		69.5	42.2	109.4	35.5	0.01
Thiamine (mg)	1.1	1.2		1.5	1.1–2.4	1.6	1.6–2.8	<0.001
Riboflavin (mg)	1.1	1.3		2.1	0.3	2.6	0.4	<0.001
Niacin (mg)	14	16		20.6	3.4	27.2	6.8	0.003
Pantothenic acid (mg)	5	5		5.7	1.1	7.9	0.8	<0.001
Vitamin B6 (mg)	1.5	1.7		2.0	0.4	2.6	0.6	<0.05
Folate (μg)	400	400		274.0	74.6	334.2	32.4	<0.05
Choline (mg)	425	550		296	149	458	95	<0.05
Vitamin B12 (μg)	2.4	2.4		4.8	3.4–6.6	6.2	6.1–7.0	<0.001
Vitamin A (IU)	700	900		8,564	9,390	21,258	7,305	<0.001
Vitamin E (mg)	15	15		3.5	1.3	5.2	1.1	0.3
Vitamin D (μg)	15	15		3.9	1.3	4.7	0.9	<0.001
Vitamin K (μg)	90	120		59.3	65.4	111.5	22.5	<0.05

*The RDA for leucine is 42 mg/kg. For a 70 kg person the respective RDA for leucine is 2,940 mg (2.9 g).

## Discussion

In the present work the provided hospital food is presented and analyzed. Indeed, when our hospital became a “COVID hospital” several changes in the standard menu were made, which led to an increase in the provision of energy, macronutrients, and several micronutrients. The total protein as well as leucine provided were increased, with changes being more evident for breakfast. This increase in nutrients provided is of outmost importance, since the dietary status is directly connected to disease severity and length of stay (7–9).

Indeed, COVID-19 patients have increased energy needs, because of fever and respiratory distress and ESPEN recommends 27–30 Kcal/kg (3). The provided menu could thus cover the energy needs of a 70 kg person (calculated needs 2,100 Kcal) since its energy content was 2,489 ± 137 Kcal. However, the estimation of real energy requirements is rather challenging in these patients and indirect calorimetry may be useful for a more accurate determination (25). In general, the energy content of the menus was higher than that described in the literature (14, 26, 27).

Another problem observed in COVID-19 patients is the fact that isolation and inactivity may lead to sarcopenia (10, 11), which can further deteriorate lung function since it depends on muscle strength (28). In order to minimize sarcopenia risk, adequate protein intake should be administrated (29). In the proposed menu more than 1 g/kg of protein are provided for a 70 kg person and higher than at least 1 g/kg, which is recommended (3). Moreover, there was an improvement in the timing of protein intake, especially in breakfast. Providing 20–30?g/meal during the three main courses (and especially from rich leucine protein sources), like in the present study ensures the proper “protein threshold” to prevent sarcopenia (30). As far as the percentage of macronutrient content of the diet is concerned, no major changes were observed. The recommended energy ratio from fat and carbohydrates is from 30:70 for subjects with no respiratory deficiency, to 50:50 for ventilated patients, i.e., 0.42–1 correspondingly (3). The provided menus both pre- and during COVID-19 had an energy ratio from fat and carbohydrate equal to 0.51, which is in line with the above recommendations (3). The addition of pudding led to an increase in simple sugars in the COVID-19 menu. This could render carbohydrate metabolism more difficult but in several clinical cases of pulmonary disease energy coverage comes first, and easily chewed foods are chosen even if they are low in complex carbohydrates (31).

Several micronutrients (vitamin A, vitamin C, vitamin E, vitamin D, selenium, omega-3 fatty acids, and minerals) have been proposed to play a role in COVID-19 and its accompanying pathophysiological effects, such as inflammation and thrombosis (2). In addition, the recommended dietary allowances (RDA) should be covered (3). In the provided menus the mean provision of most nutrients was above the RDA or adequate intake, as recommended by the Institute of Medicine, with the exception of manganese, folate, choline, and vitamins E and D (32–36). The low content of hospital diets in vitamin E and folate has been also previously underlined (37, 38). It is noted that the vitamin A content of the menus is above the recommendations. From a closer look to our data, it seems that the increased vitamin A content of the menus derives from carrots (ingredient in sauces and in salads). Indeed, 100 g carrot in the salad “cabbage with carrot,” which is the main salad used at the COVID-19 menu, contains 16,700 IU of vitamin A (NDB number at the USDA database:11124) (22). This value, however, is “calculated” in the USDA database by using conversion factors of carotenoids to vitamin A (22). The “calculated” yielding vitamin A from carrots may currently be overestimated according to the data of Tang et al. who used intrinsically deuterated vegetables to investigate the bioavailability of carrot carotenoids (39). Moreover, the toxicity of carotenoids is low (40), so even if the amounts given are above the recommendations, there is no danger for patients.

Water bottles were provided to COVID-19 patients to ensure proper hydration, providing 1.5 L of water/day. Access to additional water was possible upon request. Indeed, water intake and hydration status constitute a key weapon in the management of disease and sub-hydration has been proposed to favor fluid accumulation in the lungs (41, 42).

It is noted that both the standard and the COVID-19 menu did not fully comply with the Greek National Dietary Guidelines for healthy adults (24). In the hospital environment this chasm is inevitable since the goals for hospitalized patients are different from those for the general population (43). More particularly, for the general population we need to prevent chronic diseases, such as cancer and cardiovascular disease in the long run. For hospitalized patients (COVID-19 or non-COVID-19) we need to directly support them and cover their usually increased energy and protein needs. That is the reason why protein-rich food groups (such as meat, dairy, and eggs) are highly represented in the formulated menus. Moreover, the hospital menus have a relatively low fiber content in order not to irritate the gastrointestinal tract and to avoid increased satiety, which could further reduce food intake (44). This in part explains the low content of the hospital menus in fruits and vegetables as well as their zero content in legumes. By comparing the menu for COVID-19 patients with general (24) and disease specific recommendations (3) is clear that the menu has less adherence to national guidelines but a greater attention was given to the nutritional needs of the patients affected by COVID-19 (energy, carbohydrates, micronutrients, fluids, digestibility of the meal, lower insoluble fiber content and palatability).

In the strengths of the present work, the accuracy in the nutrient content of the menus is included. All foods are cooked in the hospital’s kitchen and the quantities of used ingredients as well as recipes of the provided dishes are known. Moreover, the main components of cooked meals were weighted to ensure consistency with the estimated cooked weights of foods. However, no chemical analysis of dishes was performed. In general, many patients do not rely totally on the hospital food (14). In the case of COVID-19, however, patients have limited access to foods and water since visitors are not allowed (13), which renders the significance of the hospital items served even greater.

Along with the interpretation of our results several points need consideration. We used the USDA database, which is based on US products (22) and several differences in micronutrients may exist regarding fortified foods, such as products which are fortified with folic acid and other B-vitamins (45). This issue mostly applies to bakery products, such as bread, since flour is enriched in the US (45). However, the provided bakery products did not change between the two investigated periods. We have analyzed the provided food using food databases, but we have not measured the actual food intake of patients, which may depend on multiple factors (15). Indeed, our experience from the Nutrition Day project suggests that a large proportion of the food remains unconsumed (46). The measured food waste in our department reaches 220 Kg per day (mean value of 40 days in the pre-COVID-19 period). However, we have no food waste data for the COVID-19 period, since a different route of food waste management was followed. Indeed, food waste from COVID-19 patients is considered as infectious waste and it was not separately weighted. Thus, the provision of hospital foods was increased but the intake of food was not measured. In addition, in the COVID-19 period the way of serving foods was also modified. The food was served in biodegradable and disposable plates and bowls, which may also contribute to reduced intake. Although the actual food intake was not recorded, it is noted that a surplus in energy and protein was provided, which ensures that even if the patient does not consume the whole meal he/she has more probability in achieving the recommendations (3). It is noted that oral nutritional supplements were also provided to COVID-19 patients on an individualized basis, as suggested by the official recommendations (3). The consumption of energy dense foods and liquids is of great importance especially in case of low oral food intake (47). This means that the actual intake of patients may be different from that provided by the basic menu. It is underlined that the presented menu should not be considered as a gold standard or guideline, but as an effort to fulfill patients’ needs in a state hospital with limited resources. Finally, the focus of the present study was mainly on the menu provided to COVID-19 patients and currently there are no data on hospitalization time, or avoidance of ICU to investigate the clinical implications of the changes made. Although the menu changes coincided with the vaccination strategy in our country (end of March 2021) making clinical correlates risky, changes in such endpoints deserve a special consideration in future studies.

In conclusion, simple menu changes and addition of comfort foods can substantially boost the nutrient content of a hospital diet, which in concert with provision of oral nutritional supplements could have an impact on patients’ nutritional status.

## Data Availability Statement

The raw data supporting the conclusions of this article will be made available by the authors upon request.

## Author Contributions

PD conceived the idea, reviewed nutrient content of the menus, undertook the statistical analysis, and wrote the manuscript. ZA-K analyzed the menus as a task for her practical placement. GK drafted the manuscript and critically reviewed the manuscript. VP undertook the statistical analysis and critically reviewed the manuscript. All authors read and approved the final manuscript.

## Conflict of Interest

The authors declare that the research was conducted in the absence of any commercial or financial relationships that could be construed as a potential conflict of interest.

## Publisher’s Note

All claims expressed in this article are solely those of the authors and do not necessarily represent those of their affiliated organizations, or those of the publisher, the editors and the reviewers. Any product that may be evaluated in this article, or claim that may be made by its manufacturer, is not guaranteed or endorsed by the publisher.
